# A strong effect of individual compliance with mass drug administration for lymphatic filariasis on sustained clearance of soil-transmitted helminth infections

**DOI:** 10.1186/s13071-021-04814-2

**Published:** 2021-06-08

**Authors:** Jérémy T. Campillo, Naomi P. Awaca-Uvon, Jean-Paul Tambwe, Godefroy Kuyangisa-Simuna, Johnny Vlaminck, Gary J. Weil, Michel Boussinesq, Cédric B. Chesnais, Sébastien D. S. Pion

**Affiliations:** 1 TransVIHMI, Université Montpellier, Institut de Recherche pour le Développement (IRD), INSERM, Montpellier, France; 2 Ministère de La Santé Publique, Kinshasa, Democratic Republic of the Congo; 3 Department of Virology, Parasitology and Immunology, Ghent University, Merelbeke, Belgium; 4 Washington University School of Medicine, St. Louis, MO USA

**Keywords:** Soil-transmitted helminths, Albendazole, Parametric survival analysis, Treatment adherence, Mass drug administration

## Abstract

**Background:**

The impact of semiannual mass drug administration (MDA) with albendazole (ALB; 400 mg) alone on lymphatic filariasis (LF) and soil-transmitted helminth (STH) infections was assessed during two trials conducted from 2012 to 2018 in the Republic of Congo and the Democratic Republic of Congo. The collected data were analyzed to evaluate the effect of compliance with ALB treatment on STH infections.

**Methods:**

STH infections were diagnosed with duplicate Kato-Katz thick smears and the results are reported as eggs per gram of stool. All subjects with at least two STH infection assessments were included in the analyses. We used parametric survival models to assess the influence of compliance with ALB treatment on the probability of (i) achieving sustained clearance of an STH infection, and (ii) acquiring an STH infection during the follow-up.

**Results:**

Out of 2658 subjects included in the trials, data on 202 participants (701 person-years; PY) with hookworm infection, 211 (651 PY) with *Ascaris lumbricoides* infection and 270 (1013 PY) with *Trichuris trichiura* infection were available to calculate the probability of achieving sustained clearance of infection. The effect of ALB was dose related for all three STH. For hookworm, the time required for sustained clearance was longer (4.3 years, *P* < 0.001) for participants who took zero doses per year and shorter (3.4 years, *P* = 0.112) for participants who took two doses per year compared to those who took one dose per year (3.7 years). For *Ascaris*, the time required to obtain sustained clearance followed the same pattern: 6.1 years (*P* < 0.001) and 3.2 years (*P* = 0.004)* vs* 3.6 years for, zero, two and one dose per year, respectively. For *Trichuris*, less time was required for sustained clearance (4.2 years, *P* < 0.001) for fully compliant participants, i.e. those who took two doses per year, than for those who only took one dose per year (5.0 years). ALB was more effective in achieving sustained clearance of STH infection in subjects with light baseline infection intensities compared to those with higher egg counts.

**Conclusion:**

Our results illustrate the importance of MDA compliance at the level of the individual with respect to the STH benefit provided by semiannual ALB MDA, which is used for the elimination of LF in Central Africa.

**Graphic abstract:**

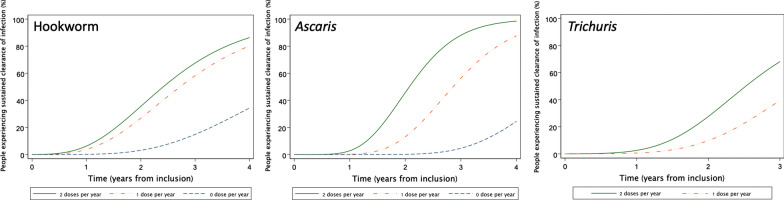

**Supplementary Information:**

The online version contains supplementary material available at 10.1186/s13071-021-04814-2.

## Background

Soil-transmitted helminth (STH) infections are among the most common infections in the world and affect poor and disadvantaged communities, particularly in sub-Saharan Africa, Southeast Asia and Latin America. In 2010, it was estimated that 438.9 million people were infected with hookworm, 819.0 million with *Ascaris lumbricoides* and 464.6 million with *Trichuris trichiura* [1]. The current strategy used to control STH infections is to conduct periodical deworming campaigns without individual diagnosis that target at-risk populations in endemic areas (preschool children, school-age children, women of reproductive age and adults in certain high-risk occupations). The World Health Organization (WHO) recommends the use of benzimidazoles: albendazole (ALB; 400 mg) or mebendazole (500 mg). Because of logistic and cost constraints, preventive chemotherapy campaigns for STH are usually conducted once or twice per year, depending on the initial prevalence of infection with any STH [2]. However, the optimal frequency of administration to maximize impact remains a topic of discussion [3]. In this study, we had the opportunity to assess the individual effect of a semiannual ALB treatment on STH infections. The data originate from two community trials that were designed to evaluate the effect of mass drug administration (MDA) with semiannual ALB on lymphatic filariasis (LF) in two countries in central Africa. The first study was conducted in a village in the Republic of the Congo (Congo), where *A. lumbricoides* infection prevalence decreased significantly from 56.5% to 12.9% and *T. trichiura* infection prevalence decreased significantly from 78.6% to 59.4% after 7 rounds of ALB MDA with global treatment adherence between 83 and 90% [4]. The second study took place in two contiguous villages in the Democratic Republic of the Congo (DRC), where hookworm infection prevalence decreased significantly from 58.6% to 21.2% after eight rounds of ALB MDA with a global treatment adherence of between 56 and 88%; *Ascaris* and *Trichuris* infection prevalences also decreased (from 14.0% to 1,6% and from 4.1% to 2.9%, respectively) [5]. The results of these two trials are consistent with those previously observed regarding ALB efficacy: according to a meta-analysis of more than 50 clinical trials, ALB has very good efficacy for clearing hookworm infections, good efficacy for *Ascaris* infections, but only moderate efficacy for *Trichuris* infections [6].

Some studies have shown that semiannual ALB MDA is highly effective for reducing STH prevalence at the community level [4, 5, 7–10]. However, considerable heterogeneity has been observed at the individual level, and no prior studies have examined the impact of the compliance of individuals with MDA on their STH infections. For example, in one study, infections cleared in some individuals after a single treatment, while infections were still present in others (due to persistence or reinfection) after eight rounds of semiannual MDA [11]. In the present study, we used longitudinal treatment and parasitology data collected between 2012 and 2018 from two community MDA studies to assess relationships between MDA compliance by individuals with STH infections and their subsequent infection status. The results indicate a clear link between MDA compliance by individuals and sustained clearance of STH infections.

## Methods

### Study population

The design of the MDA studies has been described elsewhere [4, 5]. In Congo, the study was conducted from 2012 to 2015 in Seke-Pembe, a village located in Mabombo health district (Bouenza division). In the DRC, the study site consisted of two neighboring villages (Mbunkimi and Misay) located in Kwilu province, and the trial took place from 2014 to 2018. Study participants were tested for STH infections at baseline and then annually. No deworming program had ever been conducted in the two areas prior to our trials. Both studies were approved by ethics committees and administrative authorities in the respective countries. Adult participants signed an informed consent form. Participants aged < 18 years were enrolled only after verbal assent and if one parent signed a consent form.

During the course of the two trials, a total of 2658 individuals were examined at least once for LF, and a total of 1573 provided stool samples at least once.

### Assessment of STH infections

Annual parasitological assessments were performed for participants > 5 years of age. STH infections were detected by microscopic examination of stool specimens. Participants were given a 50-mL plastic stool container and asked to collect a sample of their stool in the morning. The stool specimens were collected and stored in cooling boxes and shipped within 6 h to the laboratory, where they were immediately processed or stored overnight at 6 °C. Two thick smears were prepared according to the Kato-Katz method for each stool sample [12]. Thick smears were examined by microscopy at 40× magnification, and the slides prepared from each sample were examined by two different microscopists. The arithmetic mean egg count from the two slides was calculated, and the results are expressed for each species as eggs per gram of stool (EPG).

### Drug distribution and assessment of treatment adherence

All participants were offered treatment with one ALB tablet (400 mg) that was swallowed under the direct observation of study staff. All inhabitants who had not participated in the parasitological survey or who missed testing (due to absence or refusal) were later visited at home and offered ALB treatment. All treatments were provided under the supervision of a local healthcare worker who was also responsible for conducting a population census before each semiannual MDA. Every treatment was recorded in a drug treatment register. In addition, participants were asked whether they had received ALB during the previous MDA campaign (6 months earlier) during annual parasitological surveys. Therefore, for each annual parasitological assessment, we determined if each participant had taken two, one or zero ALB treatments since the last parasitological assessment.

### Statistical analysis

The primary endpoint (i.e. event of interest) for the study was conversion in STH infection(s). This was considered separately for conversion from a positive to a negative test during follow-up (defined as the sustained clearance of infection analysis) and for conversion from negative to positive (defined as the incident infection analysis).

The sustained clearance analysis included all individuals who were positive for *A. lumbricoides*, *T. trichiura* or hookworm at the time of their first test (which was not necessarily performed during the year when the trial started at the site) who also had at least one subsequent stool sample tested for STH. Therefore, individuals who were negative at baseline and remained negative throughout their follow-up were not included in the analyses (representing 59.1%, 47.8% and 37.6% of the population with at least one follow-up test for hookworm, *Ascaris* and *Trichuris*, respectively). The numbers of excluded individuals are provided in Additional file 1: Table S1. A sensitivity analysis including data on all the participants who were positive at the time of their first test but who had become negative by their follow-up, regardless of whether they subsequently became positive again, is provided in Additional file 1: Table S2.

The incident infection analysis included all individuals who were negative for *A. lumbricoides*, *T. trichiura* or hookworm at the time of their first stool test who had a positive stool test at a later time point (regardless of whether they subsequently became negative again).

We used survival analysis methods for the sustained clearance and incident infection analyses. The start date for the survival analysis was the first visit (index date) with a positive STH test for the sustained clearance of infection analysis or a negative STH test for the incident infection analysis. Individual observations were censored at the end of the follow-up or at the date of the event (date of the annual parasitological survey). Each participant’s data were considered for calculation of cumulative person-years (PY) in the survival analysis.

We considered the following non-time-varying covariates for each STH analysis: sex and initial EPG intensity (categorized according to WHO guidelines [13]). We also considered the following time-varying covariates: age (categorized according to interquartile and median values—5–8, 9–12, 13–30 and ≥ 31 years old); and the number of ALB tablets taken during the previous year (0, 1 or 2).

Univariate analysis of infection status conversion rates was conducted using Mantel–Haenszel tests. We used parametric survival models with accelerated failure time to estimate the influence of time-varying variables on infection status conversion (time-to-event) [14, 15]. These models allow longitudinal analyses with time-varying variables; they are more informative and provide time ratios that enable prediction of mean time until an event occurs (either sustained clearance or incident infection events).

Several time distributions that do not require meeting the proportional risk assumption were tested according to the Akaike information criterion (AIC). Random effects at the household and at the village level were assessed in all survival models, and significance was assessed using the results of likelihood ratio tests. Significant random effects were retained in the models. Results are presented as time ratios with 95% confidence intervals (CI). Time ratios represent time differences to event (individual infection status conversion) according to the reference category. Sociodemographic data, occupation, initial infection intensity and the number of ALB tablets taken per year were included in the *Ascaris*, *Trichuris* and hookworm infection status conversion survival models. For the *Trichuris* model, the variable Numbers of ALB tablets taken per year had only two categories (1 or 2) because only 1 PY contributed to the zero-dose category. Predicted average times to infection status conversions were estimated using the command margins in STATA v.15.1 software (StatCorps, College Station, TX) [16].

For individuals for whom sustained clearance of infection was achieved, mixed models with random effects were used to describe changes in EPG according to time, treatment history and sociodemographic information for each STH infection. Several transformations (linear, quadratic, first-order fractional polynomials and second-order fractional polynomials) were tested for the time variable, and selection was made according to AIC. Random effects at village level were considered for the parametric survival analysis and mixed models for changes in EPG. Lastly, the significance of relevant interaction terms was assessed (age and sex, age and initial infection intensity, age and number of ALB treatments taken, sex and initial infection intensity, sex and number of ALB treatments taken) for all models. All analyses were performed using STATA v.15.1 software.

## Results

### Study participants

For the sustained clearance model, repeated observations were made for the hookworm infection analysis for 202 of 2658 participants enrolled in the studies (7.6%), with 701 PY of observations. Stool examinations were negative for hookworm for 135 of these participants (66.8% of individuals diagnosed with hookworm infection at their first parasitological exam) during the course of the study, which remained the case until their last follow-up visit. For *Ascaris*, there were longitudinal data for 211 (7.9%) participants for analysis, with 681 PY of observations. In 172 of these participants (81.5% of individuals with *Ascaris* at their first parasitological exam), stool samples became negative during the course of the study and remained so until their last follow-up visit. For *Trichuris*, there were baseline and follow-up data for 270 participants (10.2% of all study participants) for the analysis, with 1,019 PY of observations. Of these participants, 85 (31.5% of individuals with a *Trichuris* infection at their first parasitological exam) became negative during the course of the study and remained so until their last follow-up visit. The key infection survival data are summarized in Table 1 along with results of a bivariate analysis of co-factors. Of note, most individuals with hookworm infection lived in the DRC study site, whereas most individuals with *Ascaris* and *Trichuris* infections lived in the Congo study site.

**Table 1:** Sustained clearance rates for hookworm, *Ascaris* and *Trichuris* infections

Variables	Categories	Hookworm (202 individuals)					*Ascaris* (211 individuals)					*Trichuris* (270 individuals)				
		PY	No. of events	Rate^a^	95% CI	*P*	PY	No. of events	Rate^a^	95% CI	*P*	PY	No. of events	Rate^a^	95% CI	*P*
	All participants	701	135	19.2	16.2–22.8		681	172	25.2	21.7–29.3		1019	86	8.4	6.8–10.4	
Sex	Male	390	69	17.7	14.0–22.4	0.023	299	63	21.1	16.5–27.0	0.017	420	36	8.6	6.2–11.9	0.919
	Female	311	66	21.2	16.7–27.9		382	109	28.5	23.7–34.4		599	50	8.3	6.3–11.0	
Age	5–8 Years	245	30	12.2	8.6–17.5	0.009	153	20	13.1	8.4–20.3	0.001	201	7	3.5	1.7–7.3	< 0.001
	8–12 Years	166	39	23.5	17.2–32.2		189	46	24.3	18.2–32.5		222	9	4.0	2.1–7.8	
	13–30 Years	135	24	17.8	11.9–26.5		117	32	27.3	19.3–38.7		209	16	7.6	4.7–12.5	
	≥ 31 Years and more	155	42	27.1	20.0–36.6		222	74	33.3	26.5–41.9		387	54	13.9	10.7–18.2	
Initial infection intensity^b^	Light	575	131	20.0	16.8–23.7	0.059	269	77	28.6	22.9–35.8	0.022	634	74	11.7	9.3–14.7	< 0.001
	Moderate	29	4	13.8	5.2–36.5		374	87	23.3	18.8–28.7		369	11	3.0	1.6–5.4	
	Heavy	16	0	0	N/A		38	8	21.0	10.5–42.1		16	1	6.2	0.9–44.4	
Village	Misay	323	61	18.9	14.7–24.3	0.001	44	8	18.2	9.1–36.3	0.247	3	1	N/A	N/A	
	Mbunkimi	336	56	16.7	12.8–21.6		41	13	31.7	18.4–54.6		20	4	N/A	N/A	
	Seke Pembe	42	18	42.8	27.0–68.0		596	151	25.3	21.6–29.7		994	80	8.0	6.5–10.0	

*P* is calculated from significance tests using the Mantel–Haenszel method based on stratified rate ratios

*PY* Person-years,* CI* confidence interval

^a^Infection status conversion rate (for 100 PY)

^b^According to World Health Organization (WHO) guidelines: for hookworm, 1–1999 (light), 2000–3999 (moderate), > 4000 eggs per gram of stool (EPG) (heavy); for *Ascaris*, 1–4999 (light), 5000–49,999 (moderate), > 49,999 EPG (heavy); for *Trichuris*, 1–999 (light), 1000–9999 (moderate), > 10,000 EPG (heavy)

For the incident infection model, out of 542 individuals negative for hookworm at baseline with at least one follow-up visit, 102 (220 PY) experienced an incident infection (18.8%). For *Ascaris*, out of 533 participants negative at baseline with at least one follow-up visit, 177 (727 PY) experienced an incident infection (33.2%). For *Trichuris*, out of 474 participants negative at baseline with at least one follow-up visit, 194 (772 PY) experienced an incident infection (40.9%).

### Bivariate analysis of sustained clearance of STH infection

The unadjusted sustained clearance rates for hookworm, *Ascaris* and *Trichuris* infections were 19.2, 25.2, and 8.4 events per 100 PY of observation, respectively (Table 1). Table 1 also shows sustained clearance rates adjusted for each covariable. Because the number of ALB tablets taken per year is a time-varying variable, it was not included in the sustained clearance rate calculations. For hookworm and *Ascaris*, the probability of sustained clearance was higher for females than for males. Older individuals converted to negative more often than children for all three STH infections. Despite low numbers of participants with heavy worm loads in the earlier years of the study, the initial intensity of infection was negatively correlated with the probability of sustained clearance for *Ascaris* and *Trichuris*. Finally, the probability of sustained clearance varied by village of residence.

### Parametric survival multivariate models for sustained clearance of STH infection

Parametric survival model results for sustained clearance are presented in Table 2. For the hookworm and *Ascaris* models, a log-normal distribution gave the best fit to the data, whereas a log-logistic distribution was a better fit for *Trichuris*. No significant interactions between the covariates were found. Household random effects were included for the *Trichuris* model [intraclass correlation coefficient (ICC) 12.2%, *P* = 0.002]. Village random effects were included for the hookworm model (ICC 18.0%, *P* < 0.001). Random effects were not significant for the *Ascaris* model. For hookworm, *Ascaris* and *Trichuris*, older individuals (> 30 years of age) achieved sustained clearance more rapidly than children aged 5–8 years (3.3* vs* 5.7 years for hookworm; 3.1* vs* 3.8 years for *Ascaris* and 4.1* vs* 4.7 years for *Trichuris*, respectively). It took significantly longer for non-compliant individuals (zero doses per year) to achieve sustained clearance for *Ascaris* or hookworm than for individuals who took one dose per year [6.1* vs* 3.6 years for *Ascaris* (*P* < 0.001) and 4.3* vs* 3.7 years for hookworm (*P* < 0.001), respectively]. In addition, it took less time to achieve sustained clearance of *Ascaris* and *Trichuris* infection in individuals who were highly compliant with MDA (two doses per year) compared to individuals who took only one dose per year [3.2* vs* 3.6 years for *Ascaris* (*P* = 0.004) and 4.2* vs* 5.0 years for *Trichuris* (*P* < 0.001), respectively].

**Table 2:** Results of parametric survival models for sustained clearance of hookworm, *Ascaris* and *Trichuris* infections (with random effects)

Variables	Categories	Hookworm		*Ascaris*		*Trichuris*	
		TR/95% CI^a^	*P*	TR/95% CI^a^	*P*	TR/95% CI^a^	*P*
Sex	Female	Ref.		Ref.		Ref.	
	Male	1.10 (1.01, 1.19)	0.020	1.11 (1.02, 1.21)	0.013	0.99 (0.92, 1.06)	0.807
Age	5–8 Years	Ref.		Ref.		Ref.	
	8–12 Years	0.93 (0.83, 1.04)	0.223	0.95 (0.86, 1.08)	0.398	1.06 (0.91, 1.23)	0.452
	13–30 Years	0.87 (0.77, 0.98)	0.019	0.86 (0.75, 1.00)	0.046	0.96 (0.83, 1.10)	0.557
	More than 30 years	0.79 (0.70, 0.88)	< 0.001	0.81 (0.71, 0.92)	0.001	0.88 (0.77, 0.99)	0.049
Initial infection intensity^b^	Light	Ref.		Ref.		Ref.	
	Moderate to heavy	1.20 (0.98, 1.46)	0.072	1.06 (0.97, 1.16)	0.170	1.18 (1.07, 1.31)	< 0.001
Treatment	Zero dose per year	1.16 (1.00, 1.35)	< 0.001	1.74 (1.28, 2.38)	< 0.001	Not calculable	
	One dose per year	Ref.		Ref.		Ref.	
	Two doses per year	0.91 (0.81, 1.02)	0.112	0.87 (0.79, 0.95)	0.004	0.84 (0.77, 0.91)	< 0.001
Random effects		Village	< 0.001	Not included		Household	0.002
	ICC	18.0%				12.2%	
Model	Distribution	Log normal		Log normal		Log logistic	
	AIC	464.1		515.9		410.2	
	Log likelihood	− 222.0		− 249.0		− 196.1	

*Ref.* Reference, *ICC* intraclass correlation coefficient, *AIC* Akaike information criterion; for other abbreviations, see Table 1

^a^Adjusted time ratio (*TR*)/95% confidence intervals (*CI*). For example, for the *Ascaris* model, compared to a female (TR = 1), it took 11% (TR = 1.11) more time to achieve sustained clearance in a male

^b^According to WHO guidelines: for hookworm, 1–1999 (light), > 2000 EPG (moderate to heavy); for *Ascaris*, 1–4999 (light), > 5000 EPG (moderate to heavy); for *Trichuris*, 1–999 (light), > 1000 EPG (moderate to heavy)

**Fig. 1:**
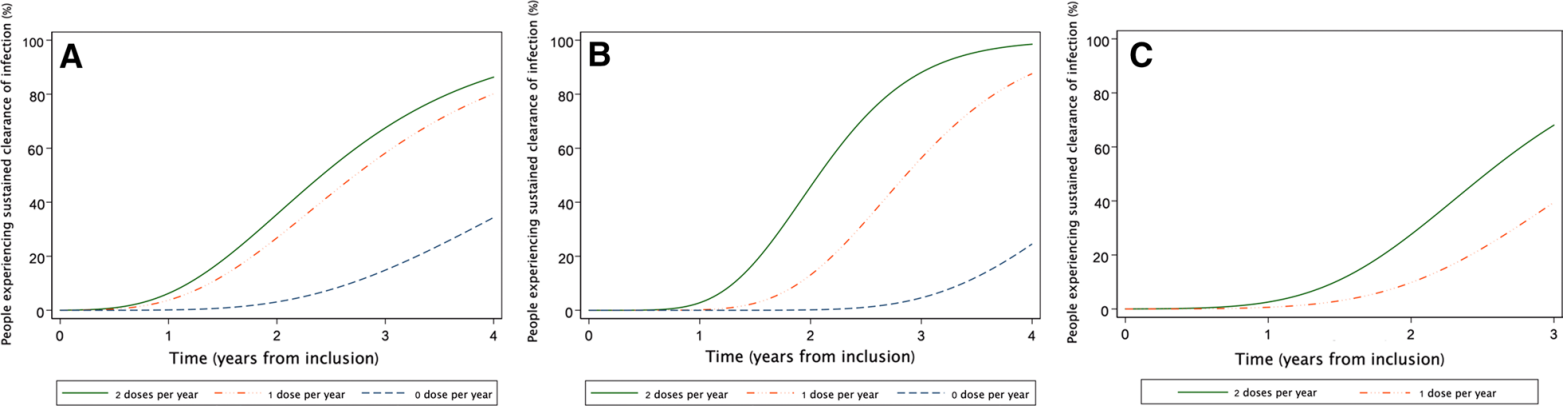
**a–c** The influence of compliance with albendazole treatment on soil-transmitted helminth infection status in study participants with prior infections. **a** Hookworm, **b** *Ascaris*, **c** *Trichuris*

Figure 1 illustrates that, for all three STH infections, the predicted proportion of participants who experienced sustained clearance of infection was higher in highly compliant individuals and increased with the duration of follow-up. Figure 1 also shows that in individuals with low compliance, sustained clearance was observed after 2 and 3 years of follow-up for hookworm and *Ascaris* infection, respectively, whereas this started after 1 year in fully compliant individuals.

Regarding infection intensity, it took significantly longer to achieve sustained clearance in individuals with moderate to heavy initial infection intensity with *Trichuris* (≥ 1000 EPG) than in individuals with light infections (< 1000 EPG) (4.2* vs* 5.0 years, respectively). Baseline infection intensities did not significantly affect the time to sustained clearance for *Ascaris* or hookworm infections.

### Parametric survival multivariate models of STH incident infection

Parametric survival model results for incident infection are presented in Table 3. A log-logistic distribution gave the best fit for data in the hookworm and *Trichuris* models; a log-normal distribution provided the best fit for the *Ascaris* model. No interactions between the covariates were found. Household random effects were included for the *Trichuris* model (ICC 42.9%, *P* < 0.001). Village random effects were included for the *Ascaris* models (ICC 45.7%, *P* < 0.001) and hookworm model (ICC 31.4%, *P* < 0.001). For hookworm and *Trichuris*, males acquired infection significantly more slowly than females. For the *Trichuris* model, older individuals (> 13 years) acquired infection significantly faster than individuals aged 5–8 years. Non-compliant individuals (zero doses per year) acquired *Ascaris* infection significantly more quickly than individuals who took one dose of ALB per year.

**Table 3:** Results of parametric survival models for incident hookworm, *Ascaris* and *Trichuris* infections (with random effects)

Variables	Categories	Hookworm		*Ascaris*		*Trichuris*	
		TR/95% CI	*P*	TR/95% CI	*P*	TR/95% CI	*P*
Sex	Female	Ref.		Ref.		Ref.	
	Male	1.39 (1.11, 1.73)	0.004	1.10 (0.94, 1.19)	0.234	1.22 (1.031, 1.44)	0.018
Age	5–8 Years	Ref.		Ref.		Ref.	
	8–12 Years	1.12 (0.87, 1.45)	0.361	1.07 (0.88, 1.32)	0.489	0.88 (0.73, 1.05)	0.156
	13–30 Years	1.02 (0.72, 1.46)	0.872	1.13 (0.87, 1.46)	0.364	0.62 (0.46, 0.84)	0.002
	More than 30 years	0.95 (0.70, 1.28)	0.738	1.11 (0.87, 1.41)	0.412	0.53 (0.40, 0.70)	< 0.001
Treatment	Zero doses per year	0.79 (0.51, 1.22)	0.290	0.62 (0.42, 0.91)	0.001	0.78 (0.53, 1.13)	0.190
	One dose per year	Ref.		Ref.		Ref.	
	Two doses per year	0.97 (0.70, 1.33)	0.828	1.10 (0.80, 1.50)	0.559	1.14 (0.85, 1.54)	0.385
Random effects		Village	< 0.001	Village	< 0.001	Household	< 0.001
	ICC	31.4%		45.7%		42.9%	
Model	Distribution	Log logistic		Log normal		Log logistic	
	AIC	505.5		626.5		718.4	
	Log likelihood	− 243.7		− 304.2		− 350.2	
Key survival data	Number of subjects	102		177		194	
	PY	420		727		772	

For abbreviations, see Tables 1 and 2

### Changes in EPG over time

Linear transformation of time provided the best fit for the EPG data. No multilevel effects were included in these mixed models because neither the village nor the household effect was significant. Overall decreases in EPG were superior in individuals with better compliance (Table 4). For all three STH, the decline in EPG over time was slower in younger individuals, regardless of the number of ALB tablets taken per year.

**Table 4:** Mixed model results for the evolution of EPG intensity

Variables	Categories	Hookworm		*Ascaris*		*Trichuris*	
		Coeff./95% CI^a^	*P*	Coeff./95% CI^a^	*P*	Coeff./95% CI^a^	*P*
Sex	Female	Ref.		Ref.		Ref.	
	Male	65.8 (− 34.4, 166.0)	0.199	1056.7 (− 1115.9, 3229.2)	0.340	234.2 (− 139.1, 607.5)	0.219
Age	5–8 Years	Ref.		Ref.		Ref.	
	8–12 Years	32.6 (− 122.6, 187.8)	0.681	− 4540.2 (− 7576.5, − 1504.0)	0.003	126.1 (− 439.3, 691.6)	0.662
	13–30 Years	− 128.0 (− 274.1, 18.0)	0.086	− 6432.5( − 9910.3, − 2954.8)	0.0001	− 333.9 ( − 929.6, 261.7)	0.272
	More than 30 years	− 107.1 (− 251.4, 37.1)	0.088	− 8030.3 (− 11035, − 5024.5)	< 0.001	− 498.6 (− 1179.8, − 162.3)	0.010
Initial infection intensity^b^	Light	Ref.		Ref.		Ref.	
	Moderate	1155.4 (929.3, 1383, 5)	< 0.001	8727.8 (6302.6, 11153)	< 0.001	1061.2 (653.1, 1469.4)	< 0.001
	Heavy	3171.6 (2875.8, 3467.3)	< 0.001	33, 843 (28637, 39050)	< 0.001	6886.7 (5518.5, 8254.9)	< 0.001
Time^c^	Continuous	− 58.2 (− 263.2, 146.6)	0.577	1631.5 (− 4174.5, 7437.4)	0.582	− 91.9 (− 378.8, 195.0)	0.530
Annual treatment interaction with time^c^	Zero doses	Ref.		Ref.		Not calculable	
	One dose	− 81.2 (− 316.0, 153.6)	0.498	− 7076.1 (− 13,103, − 1048.9)	0.021	Ref.	
	Two doses	− 197.1 (− 402.6, 8.4)	0.060	− 6207.3 (− 12,196, − 218.5)	0.042	− 207.5 (− 603.2, 188.3)	0.304
Study site	Bandundu	Ref.		Ref.		Ref.	
	Seke Pembe	− 196.9 (− 427.8, 34.0)	0.095	2596.8 (− 1004.8, 6198.3)	0.158	721.0 (− 410.1, 1852.1)	0.212
Intercept at baseline	Zero doses	387.4 (− 478.5, 1253.2)	0.381	− 7884 − 33,495, 17,725.7)	0.546	Not calculable	
	One dose	272.7 (− 636.0, 1181.4)	0.556	22,598 (− 3365.0, 48,561)	0.088	− 199.5 (− 170.9, 1305.9)	0.795
	Two doses	404.0 (− 439.6, 1247.7)	0.348	22,888 (− 2750.4, 48,527)	0.080	936.6 (− 192.0, 2065.1)	0.104

^a^Adjusted regression coefficient (*Coeff.*)/95% CI

^b^According to WHO guidelines: for hookworm, 1–1999 (light), 2000–3999 (moderate), > 4000 EPG (heavy); for *Ascaris*, 1–4999 (light), 5000–49,999 (moderate), > 49,999 EPG (heavy); for *Trichuris*, 1–999 (light), 1000–9999 (moderate), > 10,000 EPG (heavy)

^c^Interpretation of the interaction variable: for the hookworm model, all else being equal, each participant’s EPG decreased by 58.2 each year; and all else being equal, participants taking one dose and two doses per year, as compared to zero doses, showed a decrease in their EPG by 81.2 and 197.1 EPG per year, respectively

According to the mixed model, decreases in hookworm EPG were not significantly different for individuals with different compliance patterns: —81.2 EPG/year (95% CI − 316.0–153.6) for one dose and − 197.1 EPG/year (CI 95% − 402.6–8.4) for two doses, compared with no treatment. Decreases in *Ascaris* EPG were significantly different between individuals who took zero and one dose per year (regression coefficient: − 7076.1 EPG/year, CI 95% − 13,103 to − 1048.9) and between those who took zero and two doses per year (regression coefficient: -6,207.3 EPG/year, CI 95% − 1.2196 to − 218.5). Finally, decreases in *Trichuris* EPG were not significantly different between individuals who took two doses per year (regression coefficient: -207.5 EPG/year, CI 95% − 603.2–188.3) and those who took one dose per year.

## Discussion

ALB is widely known to be effective for the treatment of STH infections. Community MDA with ALB can reduce *Ascaris* and hookworm prevalence, but the effects on *Trichuris* tend to be modest [6, 17]. This is the first longitudinal study to examine the effect of individual compliance with semiannual MDA with ALB alone, given for the elimination of LF, on STH infections. We found that good compliance with semiannual rounds of MDA resulted in shorter times to achieve sustained clearance of STH infections. This was likely due to a combination of curing existing infections and curing incident infections during the follow-up period. This dose-related effect was particularly strong for ascariasis. A similar pattern was observed for hookworm, although the difference between one and two doses per year was not statistically significant. This might indicate that reinfection is more rapid for hookworm than for *Ascaris*. Further studies are needed to validate this hypothesis. As individuals with *Trichuris* infection in this study were generally compliant with MDA, we were unable to compare times to infection status conversion based on ALB intake. It is interesting that some non-compliant individuals with hookworm or *Ascaris* infections achieved sustained clearance, and that this phenomenon was more common in the later years of the study, as shown in Fig. 1. Although some spontaneous loss of infection was expected, particularly for light infections, an increase in sustained clearance events in later years might have been due to a reduced force of infection in study communities (due to a “herd treatment effect”) as a result of MDA.

The use of random effects at the household level improved the parametric survival model for *Trichuris*. Continued infection in households due to non-compliance or shared poor sanitation might increase the risk of reinfection for other household members who do comply with treatment. This would tend to increase the mean time for the sustained clearance of infection. Additional studies will be needed to test this hypothesis and to understand why the same household effect was not seen with hookworm or *Ascaris* infection.

It is interesting that incident infections were observed for all three STH infections during the course of the study despite community MDA. While most of these were probably true incidence events, it is likely that some of them were examples of pseudo-incidence, which may occur if light infections are not detected in prior stool samples.

Based on our analysis, good compliance with MDA was most effective for preventing *Ascaris* infections. This was because random household effects were significant for the *Trichuris* incidence model and for the hookworm incidence model. This means that a fraction of the incident infections for hookworm and *Trichuris* were due to random household effects regardless of MDA compliance. Random effects set at the village level were significant for the hookworm sustained clearance model and the *Ascaris* incident infection model. This suggests that a significant proportion of these status conversions were due to common village-specific effects related to sanitation and/or infection-promoting behavior.

Higher infection intensity was negatively associated with sustained clearance for all three STH, but this only resulted in a significantly longer time to achieve sustained clearance for *Trichuris*. However, low baseline frequencies of moderate or high infection intensities for hookworm and *Ascaris* provided low statistical power for assessing the effect of infection intensity for these infections. For all models, a shorter time to sustained clearance was associated with older age. This is consistent with higher reinfection rates in children due to their behavior and exposure (e.g. frequent close contact with other children, walking barefoot outdoors, and hand to soil to mouth) relative to those of older people.

We used mixed models to study changes in infection intensity (EPG) over time according to the number of ALB tablets taken per year for each STH. Only the *Ascaris* model showed a greater decrease in EPG for compliant participants. No significant differences were found for EPG decrease and ALB compliance in the hookworm and *Trichuris* models. This was probably due to a lack of power because EPG trended down in regression models as compliance increased.

One limitation of this study is that results may have been influenced by prevalence or participation bias. We believe that prevalence bias is unlikely to have impacted our results because fewer than 20% of the study population had taken ALB prior to our study. On the other hand, participation bias cannot be excluded because participants with a high participation frequency in our study may have had different characteristics (including MDA adherence) than non-participants.

As previously reported, MDA and stool survey participation rates decreased over time in these community MDA studies [5]. This was probably due to study fatigue on the part of some community members. The results of this study that show the clear benefits of compliance with MDA may help social mobilization campaigns to increase initial and sustained MDA adherence by individuals and populations.

## Conclusions

This study demonstrated a clear dose–response relationship between individual MDA adherence with semiannual ALB and sustained clearance of STH infection over time. In other words, sustained clearance of STH infections was more likely to be achieved in individuals with good MDA adherence. These results should be used in social mobilization programs to publicize the fact that semiannual MDA with ALB for the elimination of LF also significantly contributes to the control of STH infections in individuals and communities.

## Supplementary Information

**Additional file 1: Table S1. **Individuals included or not included in the sustained clearance analysis.* DRC* Democratic Republic of the Congo,* Congo* Republic of the Congo. **Table S2. **Sensitivity analysis including data on participants who were positive at the time of their inclusion in the study and whose status sequentially changed to negative and to positive again during their follow-up.

## Data Availability

The datasets used and/or analyzed during the current study are available from the corresponding author on request.

## Abbreviations

AIC: Akaike information criterion
ALB: Albendazole
CI: Confidence interval
DRC: Democratic Republic of the Congo
EPG: Eggs per gram of stool
ICC: Intraclass correlation coefficient
LF: Lymphatic filariasis
MDA: Mass drug administration
PY: Person-years
STH: Soil-transmitted helminth
WHO: World Health Organization
