# Supplementary material for: A strong effect of individual compliance with mass drug administration for lymphatic filariasis on sustained clearance of soil-transmitted helminth infections
Source: Parasit Vectors. 2021 Jun 8;14:310. doi: 10.1186/s13071-021-04814-2 (PMC8186172; doi:10.1186/s13071-021-04814-2)
Supplement: Supplementary file 1 — Additional file 1: Table S1. Individuals included or not included in the sustained clearance analysis. DRC Democratic Republic of the Congo, Congo Republic of the Congo. Table S2. Sensitivity analysis including data on participants who were positive at the time of their inclusion in the study and whose status sequentially changed to negative and to positive again during their follow-up. [file 13071_2021_4814_MOESM1_ESM.docx]

|  | **Hookworm infection** | | ***Ascaris* infection** | | ***Trichuris* infection** | |
| --- | --- | --- | --- | --- | --- | --- |
|  | **DRC** | **Congo** | **DRC** | **Congo** | **DRC** | **Congo** |
| **Number of participants included in the sustained clearance analysis** | **202** | | **211** | | **270** | |
| - Participants who had sustained clearance of their infections | 86 | 18 | 21 | 151 | 5 | 80 |
| - Participants who did not clear their infections | 98 | 0 | 6 | 33 | 3 | 182 |
| **Number of participants not included in the sustained clearance analysis** | **2456** | | **2447** | | **2388** | |
| - No follow-up (only 1 visit) | 911 | 1003 | 911 | 1003 | 911 | 1003 |
| - Negative at all points | 81 | 359 | 207 | 149 | 217 | 63 |
| - Progressed from negative to positive test | 101 | 1 | 132 | 45 | 141 | 53 |

**Additional file 1: Table S1. Individuals included or not included in the sustained clearance analysis.**

DRC, Democratic Republic of the Congo; Congo, Republic of the Congo

| **Variables** | **Categories** | **Hookworm (209 individuals)** | | ***Ascaris* (222 individuals)** | | ***Trichuris* (285 individuals)** | |
| --- | --- | --- | --- | --- | --- | --- | --- |
|  |  | **TR / 95% CI ^a^** | ***P*** | **TR / 95% CI ^a^** | ***P*** | **TR / 95% CI ^a^** | ***P*** |
| Sex | Female | Ref. |  | Ref. |  | Ref. |  |
|  | Male | 1.12 [1.03,1.21] | 0.005 | 1.10 [1.01,1.19] | 0.022 | 0.98 [0.91,1.06] | 0.665 |
| Age | 5 – 8 years | Ref. |  | Ref. |  | Ref. |  |
|  | 8 – 12 years | 0.98 [0.88,1.09] | 0.741 | 0.96 [0.84,1.08] | 0.499 | 1.06 [0.92,1.23] | 0.397 |
|  | 13 – 30 years | 0.92 [0.80,1.05] | 0.210 | 0.89 [0.77,1.02] | 0.091 | 0.99 [0.86,1.14] | 0.867 |
|  | More than 30 years | 0.82 [0.71,0.95] | 0.009 | 0.84 [0.74,0.95] | 0.006 | 0.88 [0.77,0.99] | 0.043 |
| Initial infection intensity ^b^ | Light | Ref. |  | Ref. |  | Ref. |  |
|  | Moderate to heavy | 1.08 [0.91,1.28] | 0.396 | 1.08 [1.00,1.18] | 0.060 | 1.17 [1.06,1.29] | 0.002 |
| Treatment | 0 dose / year | 1.19 [1.02,1.38] | 0.026 | 1.77 [1.29,2.41] | <0.001 | Not calculable |  |
|  | 1 dose / year | Ref. |  | Ref. |  | Ref. |  |
|  | 2 doses / year | 0.93 [0.82,1.04] | 0.212 | 0.86 [0.79,0.95] | 0.002 | 0.80 [0.74,0.86] | <0.001 |
| Random effects |  | Household | 0.001 | Not included |  | Household | 0.004 |
|  | ICC | 15.2% |  |  |  | 11.9% |  |
| Model | Distribution | Log-normal |  | Log-normal |  | Log-logistic |  |
|  | AIC | 494.2 |  | 546.0 |  | 477.2 |  |
|  | Log likelihood | -233.1 |  | -264.0 |  | -229.6 |  |

**Table S2. Sensitivity analysis including people who were positive at the time of their inclusion and who sequentially changed to negative and to positive again during their follow-up.**

^a^ Adjusted Time Ratio / 95% Confidence intervals. For example, for the Hookworm model, compared females (TR = 1), Males took 12% (TR = 1.12) longer to achieve sustained clearance of infection. .

^b^ According to OMS guidelines (light, moderate + heavy)

- For hookworm: 1-1999, more than 2000 Eggs Per Gram (EPG)
- For *Ascaris*: 1-4999, more than 5000 EPG.
- For *Trichuris*: 1-999, more than 1000 EPG.
